# Phylogenomics of novel clones of *Aeromonas veronii* recovered from a freshwater lake reveals unique biosynthetic gene clusters

**DOI:** 10.1128/spectrum.01171-24

**Published:** 2024-11-08

**Authors:** Opeyemi U. Lawal, Noah Bryan, Valeria R. Parreira, Rebecca Anderson, Yanhong Chen, Melinda Precious, Lawrence Goodridge

**Affiliations:** 1 Canadian Research Institute for Food Safety (CRIFS), University of Guelph, Guelph, Ontario, Canada; 2 Bayview Secondary School, Richmond Hill, Ontario, Canada; USDA-ARS National Center for Cool and Cold Water Aquaculture, Kearneysville, West Virginia, USA

**Keywords:** *Aeromonas*, genomics, antimicrobial resistance, global population structure, biosynthetic gene cluster, water quality, freshwater lake, public health

## Abstract

Aquatic ecosystems serve as crucial reservoirs for pathogens and antimicrobial resistance genes, thus presenting a significant global health risk. Here, we investigated the phylogenomics of *Aeromonas veronii* from Lake Wilcox in Ontario. Among the 11 bacterial isolates, nine were identified as *A. veronii*. Notably, 67% of *A. veronii* isolates were potential human pathogens. Considerable genetic diversity was noted among the *A. veronii* isolates, suggesting the lake as a reservoir for multiple human pathogenic strains. Comparison of the *A. veronii* sequenced with global *A. veronii* genomes highlighted significant genetic diversity and suggests widespread dissemination of strains. All the isolates carried chromosomal genes encoding resistance to β-lactams. Although virulence gene content differed between human and non-human pathogenic strains, type III secretion systems was associated with human pathogenic isolates. The assessment of AMR genes in global isolates showed that β-lactam and tetracycline resistance genes were predominant. Although the machine learning-based pangenome-wide association approach performed did not yield any source-based genes, some genes were enriched in a few isolates from different sources. The *mrkABCDF* operon that mediates biofilm formation and genes encoding resistance to colistin, chloramphenicol, trimethoprim, and tetracycline were enriched in animal products, whereas macrolide resistance genes and Inc plasmid-types were linked to the aquatic environment. Novel biosynthetic gene clusters were identified, suggesting that *A. veronii* with varying pathogenic potential could produce unique secondary metabolites. There is a need for continuous tracking of pathogens in aquatic ecosystems to contribute to our understanding of their evolutionary dynamics and the ecological roles of their genetic elements.

**IMPORTANCE:**

Lakes and other aquatic ecosystems can harbor harmful bacteria that can make people sick and resist antibiotics, posing a significant global health risk. In this study, we investigated *Aeromonas veronii*, a Gram-negative bacteria found in Lake Wilcox in Ontario. We used various techniques, including whole-genome sequencing (WGS), to analyze the bacteria and found that many of the isolates had the potential to cause human disease. We also discovered significant genetic diversity among the isolates, indicating that the lake may be a reservoir for multiple human pathogenic strains. All isolates carried genes that confer resistance to antibiotics, and some virulence genes were associated with human pathogenic isolates. This study highlights the importance of monitoring aquatic ecosystems for harmful bacteria to better understand their evolution, potential for human pathogenicity, and the ecological roles of their genetic elements. This knowledge can inform strategies for preventing the spread of antibiotic-resistant bacteria and protecting public health.

## INTRODUCTION

The role of aquatic ecosystems as reservoirs for clinically relevant pathogens and antimicrobial resistance genes (ARG) has recently gained attention as the importance of assessing the quality of these ecosystem is paramount in public health (1, 2). Freshwater bodies like lakes and rivers used for recreational purposes can significantly impact the health of communities (3, 4). Poor water quality in these settings poses a substantial risk for the transmission of various waterborne diseases, including pathogenic viruses, protozoa, and bacteria including *Aeromonas* species that thrive in such contaminated water (2–4).

*Aeromonas* species are Gram-negative, facultative anaerobic rods, found in various aquatic environments (5–7), and known for their ability to survive in diverse environments, ranging from freshwater to the intestinal tracts of animals (5, 7). Although some *Aeromonas* species including *Aeromonas salmonicida*, *Aeromonas hydrophila*, and *Aeromonas veronii* are known fish pathogens, *A. veronii* is one of the four species that are considered as potential human pathogens (8–10). *A. veronii* is an emerging human pathogen causing a wide range of diseases in human and animals including gastroenteritis, respiratory and skin infections, and septicemia (9–11). In addition, *A. veronii* is increasingly being recognized as a significant concern to food safety due to its frequent presence in different types of food, particularly in minimally processed ready-to-eat seafood (12, 13). Of note, the frequent and global occurrence of highly virulent strains of *A. veronii* has been detected in food samples such as meat, milk, catfish, and fish in countries including Brazil (13), Egypt (14), India (15), Israel (11), and the USA (16, 17), among others. The adaptability of *A. veronii* to various conditions poses a challenge for water quality management, especially in environments with high anthropogenic activities, where the bacterium can be a potential source of infection (5, 18).

The mechanisms of pathogenicity of *A. veronii* involve the production of various toxins and virulence factors that contribute to its ability to infect host cells and cause disease (5, 18). A significant concern with *A. veronii* is its capacity for antimicrobial resistance (AMR) (7, 19, 20). The presence of antimicrobial-resistant strains in aquatic environments is a public health concern, as it not only affects the treatment of *Aeromonas*-related infections but also represents a potential reservoir for the spread of resistance genes to other pathogenic bacteria (7, 19, 20). Studies on the population structure of *A. veronii* have described genetic diversity driven by its adaptability to various environmental conditions. These factors could drive variability in strains regarding pathogenicity and resistance to environmental stresses in this bacterium, with practical implications for public health and water management (5, 8, 18).

In recent years, advancements in sequencing technologies have greatly enhanced the genomic surveillance of known and emerging pathogens, such as *A. veronii*, across different environmental matrices (5). Despite these technological advancements, little importance has been given to *A. veronii,* especially in terms of its presence in freshwater, its impact on water quality, and its role in the dissemination of AMR in both the environment and the food chain. Understanding the genomic surveillance and population structure of this bacterium is crucial for developing effective infection treatment strategies and ensuring public health safety.

We have previously reported the detection of clinically relevant pathogens in Lake Wilcox, including novel strains of *Bacillus anthracis* (21) and *Vibrio cholerae* (22) isolated at different time points. In this study, we employed a combination of culture-based detection and whole-genome sequencing (WGS) to assess the presence of *A. veronii*, its extensive genomic fingerprint, its population structure, and the genomic characterization of stress response genes in Lake Wilcox. The genetic relatedness of *A. veronii* isolates was assessed by comparing them with previously sequenced strains in public databases using a comparative genomic approach.

## MATERIALS AND METHODS

### Description of sampling site

Lake Wilcox is a small kettle lake located in Richmond Hill in Ontario (43°56′56.69″ N, 79°26′9.45″ W). Historically, the lake is used for recreational purposes by the surrounding community and tourists. Despite being impacted by feces of surrounding wildlife, recreational activities have continued, and users have reported skin rashes and gastrointestinal symptoms after recreational activities (https://projectboard.world/ysc/project/the-phage-takes-centre-stage-for-water-quality-testing).

### Sample collection and processing

Freshwater samples were obtained from Lake Wilcox in the summer of 2022 and fall of 2023. Water samples were kept at 4°C and analyzed within 48 h of collection. Samples were processed as described by Bryan et al. (23). Briefly, 1 mL of samples was serially diluted in 9 mL of lambda buffer (modified saline-magnesium buffer without gelatin) and plated onto tryptic soy agar (TSA). Following incubation for 24 h at 37°C, plates were analyzed for bacterial colonies. Distinct colonies of differing morphologies were sub-cultured onto TSA to obtain pure culture. The isolated colonies were Gram stained, and taxonomic identification was performed using VITEK (bioMérieux, Inc, Canada).

### Genomic DNA extraction and whole-genome sequencing

Genomic DNA from isolated colonies was extracted using the DNeasy blood and tissue kit (Qiagen, Hilden, Germany) according to the manufacturer’s instructions. DNA libraries were prepared using the Illumina DNA prep tagmentation kit (#20018704) and IDT for Illumina DNA/RNA UD indexes (#20027213) following the manufacturer’s instructions. Paired-end (2 × 150 bp) sequencing was performed using the high-output flow cell on the Illumina MiniSeq instrument as described previously (23, 24).

### Genome assembly and annotation

Raw paired-end reads were quality filtered using FastQC v0.11.9 (https://github.com/s-andrews/FastQC) and trimmed using Trimmomatic v0.39 (25). High-quality reads with a Phred quality score above 20 were assembled *de novo* using the Skesa v2.4.0 pipeline (26). Assembly quality and genome completeness were assessed using QUAST v5.2 (27) and BUSCO (28), respectively. Taxonomic classification was performed using pubMLST and rMLST (29), as well as k-*mer*-based species taxonomic classification with the Kraken2 database (30). The average nucleotide identity (ANI) analysis was performed using fastANI (31). Genome annotation was performed using Prokka v1.14.6 (32).

### Gene content analysis

The pathogenicity of the isolates was determined using the PathogenFinder tool (33), a machine learning-based model that compares the whole-proteome sequences to a database composed of protein families associated with either pathogenic or non-pathogenic organisms in humans and returns with a pathogenicity score. The antimicrobial resistance gene profile of all the isolates was determined using AMRFinder Plus v3.10.45 (34) and CARD (35) databases, whereas the virulence genes were identified using the updated virulence factor database (VFDB) (36, 37), which contains all known genes reported to be associated with bacterial virulence. Genes with a threshold of >70% coverage and >90% nucleotide identity were considered to be present. To define the mobile genetic elements (MGE) of the collection, the draft genomes were screened for plasmids using MOB-suite v3.1.6 (38) with default settings. Prophage regions were detected using PHASTEST (39) and PhaBox (40). The completeness [CheckV (41)] and classification [PhaGCN (42)], as well as lifestyle [BACPHLIP (43)] and the host [CHERRY (44)] of the detected prophage sequences were determined. Intact phages were screened for tailspike proteins (TSPs) using TSPDB that contains 8,105 TSPs (45)Lawal and Goodridge (2024 Preprint). Biosynthetic gene clusters were assessed using the antiSMASH v6 pipeline (46).

### Pangenome and phylogenetic analysis

To construct the phylogeny, the pangenome of all publicly available *A. veronii* genomes was generated from the annotated genomes using Roary v3.13.0 (47), and single nucleotide polymorphisms (SNPs) within the core-genome alignment were extracted using SNP-sites v2.5.1 (48) indicating GCA_008693705.1. The concatenated core-genome-based SNPs were used to construct a phylogenetic tree using FastTree (49). The general time reversible model was performed with 1,000 bootstrap resampling for node support. Except as otherwise stated, all bioinformatics tools were executed using the default settings. Genomic features that are exclusive and/or enriched in different source groups with ≥20 genomes were determined using the pan-genome-wide association studies (pan-GWAS) approach with Scoary v1.6.16 (50) as previously described (51, 52). Genes with a Benjamini–Hochberg *P* value of <0.05 and an odds ratio of >1, with no duplicated function in the pangenome, were considered. Moreover, machine learning approach with Fisher’s exact test was used as a second approach to identify genes significantly associated with different source categories. Specifically, the differences in antimicrobial resistance, virulence genes, and mobile genetic elements across various sources were assessed with an analysis of variance (ANOVA). The data set was normalized to account for variations in sample sizes, and the statistical significance of observed differences was determined with a *P* value <0.05.

## RESULTS

### Bacterial identification and sequencing

Bacterial species were isolated from the freshwater samples recovered from a freshwater lake over a period of 1 year using the spread agar plate method. Overall, 11 distinct colonies with different morphologies were selected and further characterized using morphological, biochemical-based, and whole-genome sequencing. Taxonomic identification using the VITEK Compact system identified the colonies as *Aeromonas sobria* (*n* = 8), *A. hydrophila/punctata* (*n* = 2), and one isolate with an inconclusive result (Table 1). Sequencing of the 11 isolates yielded 1,024,248–2,725,402 paired-ended reads per isolate (Table 1). Isolates were identified as *A. veronii* (*n* = 9), *Aeromonas caviae* (*n* = 1), and *Aeromonas allosaccharophila* (*n* = 1) (Table 1). The ANI analysis with fastANI (31) using the species demarcation threshold of 95% (53) showed that the nine *A. veronii* strains had >96% ANI when *A. veronii* GCF_000820225.1 strain was used as a reference, *A. caviae* strain NB-180 had 97.9% ANI with *A. caviae* GCF_000819785.1, whereas *A. allosaccharophila* had 96.21% ANI with the reference strain *A. allosaccharophila* GCF_000819685.1. The draft genomes of *Aeromonas* species yielded between 28 and 113 contigs, with a G+C content of 58%–59%, except for *A. caviae* that had a higher G+C content of 61.26%, a value that was comparable to the reference strain *A. caviae* GCF_000819785.1. The genome size was comparable between the three *Aeromonas* species identified and ranged between 4,390,436 and 4,690,056 bp, with >50× genome coverage (Table 1).

**TABLE 1:** Summary of sequence metrics of *Aeromonas* isolates recovered from a freshwater lake

Isolate ID	Isolation period	Coverage	# Contigs	Genome size	% GC	N50	CDS	misc_RNA	rRNA	tRNA	tmRNA	Repeat region	Vitek ID	WGS-based ID	ST	Assembly accession	SRA accession
NB-178	Fall 2023	142.68	44	45,73,701	58.62	2,22,570	4,073	46	9	84	1	0	*A. sobria*	*A. veronii*	2530	GCA_039652535.1	SRR28980392
NB-180	Fall 2023	134.57	50	45,00,871	61.26	1,73,045	4,069	48	3	84	1	0	*A. hydrophila/punctata*	*A. caviae*	2531	GCA_039652415.1	SRR28980391
NB-181	Fall 2023	53.34	90	46,01,063	58.61	1,61,830	4,101	47	9	67	1	0	Inconclusive	*A. veronii*	2530	GCA_039652515.1	SRR28980390
NB-185	Fall 2023	142.75	48	46,38,531	58.75	1,76,021	4,225	50	12	76	1	0	*A. sobria*	*A. veronii*	2532	GCA_039652495.1	SRR28980389
NB-187	Fall 2023	138.89	30	43,90,436	59.02	3,00,356	3,957	51	7	2	1	2	*A. sobria*	*A. veronii*	2533	GCA_039652475.1	SRR28980388
NB-188	Fall 2023	135.78	39	46,90,056	58.54	2,38,966	4,250	51	6	78	1	0	*A. hydrophila/punctata*	*A. veronii*	2534	GCA_039652455.1	SRR28980387
NB-2	Summer 2022	73.11	46	44,65,557	59.01	2,33,461	4,074	47	7	72	1	1	*A. sobria*	*A. veronii*	2535	GCA_026571335.1	SRR22013455
NB-3	Summer 2022	88.8	28	45,93,492	58.78	4,39,357	4,135	51	6	80	1	0	*A. sobria*	*A. veronii*	2536	GCA_026571285.1	SRR22013454
NB-4	Summer 2022	97.22	44	44,66,772	59.01	1,79,674	4,071	47	7	76	1	0	*A. sobria*	*A. veronii*	2535	GCA_026571345.1	SRR22013453
NB-6	Summer 2022	94.85	53	44,83,134	58.83	2,11,391	4,052	44	12	79	1	0	*A. sobria*	*A. veronii*	2537	GCA_026571275.1	SRR22013452
NB-7	Summer 2022	75.89	113	46,08,243	58.93	97,905	4,179	43	3	83	1	1	*A. sobria*	*A. allosaccharophila*	2538	GCA_035798095.1	SRR22013451

### Prediction of human pathogenicity of *A. veronii* sequenced

Considering that *Aeromonas* species are commonly associated with diseases in fish, we evaluated the potential of these isolates to be pathogenic to humans using PathogenFinder tool (33). Six out of the nine *A. veronii* isolates sequenced in this study had a pathogenicity score greater than 0.5, suggesting that they may be pathogenic to humans. Other isolates, including *A. caviae* and *A. allosaccharophila*, were predicted to be non-human pathogens (Table 1).

### Population structure of *A. veronii* isolated from freshwater

**Fig 1:**
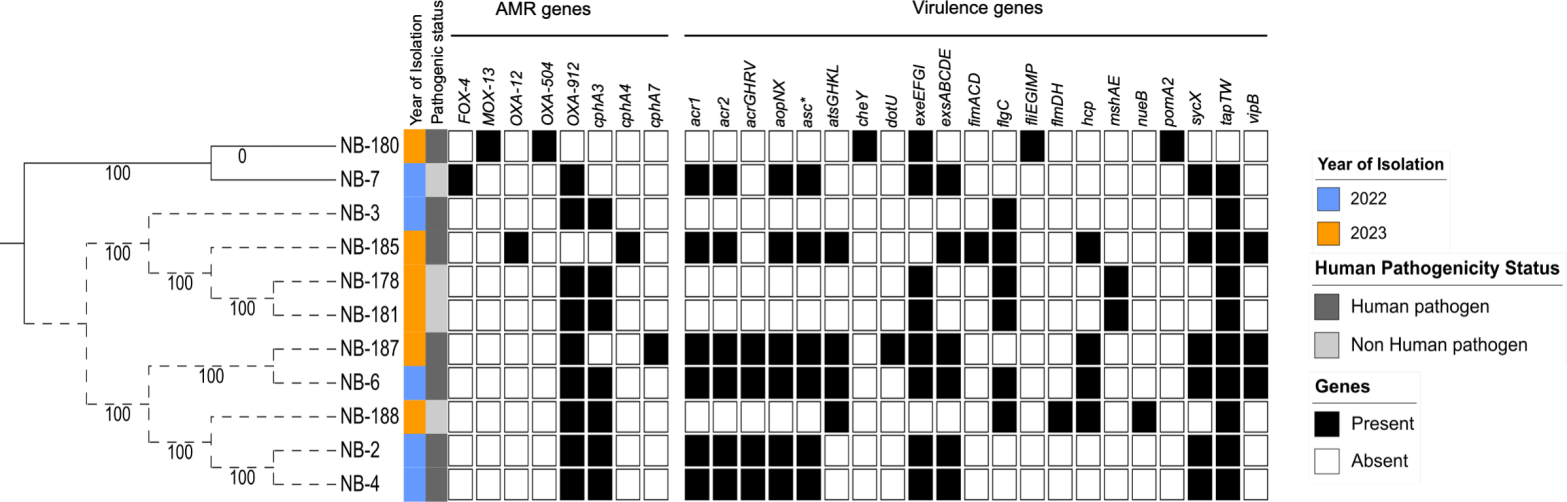
Maximum likelihood tree of *A. veronii* recovered from freshwater lake in Ontario. Each node represents a strain. To construct the phylogeny, pangenome was generated from the annotated genomes using Roary v3.13.0. SNPs within the core-genome alignment were extracted with SNP-sites v2.5.1 using GCA_008693705.1 as a reference. The concatenated core-genome-based SNPs were used to construct a phylogenetic tree using FastTree. The general time reversible model was performed with 1,000 bootstrap resampling for node support. The dotted lines depict *A*. *veronii*. NB-7 (*A. allosaccharophila*) and NB-180 (*A. caviae*) were outgroups used to re-root the tree. The draft genomes were screened for genes encoding antimicrobial resistance and virulence using CARD and VFDB databases, respectively. The tree was visualized using iTOL (https://itol.embl.de).

To assess the genetic relatedness among isolates sequenced in this study, a combination of conventional multilocus sequence typing (MLST) and whole-genome-based phylogeny was employed. The *Aeromonas* MLST schema was used to determine the sequence types (STs) of all isolates. Of note, 41 novel alleles were identified among the 11 *Aeromonas* isolates and yielded nine unique allele profiles that were submitted together with the allele sequences and assigned to nine new STs (ST2530–ST2538) (Table 1; Table S1). Two STs (ST2530 and ST2535) contained two isolates each, whereas others were singletons suggesting the uniqueness of the isolates understudy and high genetic diversity in the population. The core-genome SNP-based phylogeny of the nine *A. veronii* sequenced was constructed using the complete genome of *A. veronii* GCA_008693705.1 as a reference. *A. caviae* and *A. allosaccharophila* were used as outgroups to re-root the tree. Isolates were grouped into two main clusters irrespective of the period of isolation (Fig. 1). Isolates were distantly related by SNPs with ≥100 SNPs difference (Table S2) except for a pair of isolates from different timepoints (NB-2/NB-4, summer 2022, and NB-178/NB-181, fall 2023) that were highly related differing only by 9 and 11 SNPs, respectively (Fig. 1). Of note, the SNP-based clustering observed was similar to the MLST-based population structure suggesting a good concordance between these methods for typing *A. veronii*. Overall, the high genetic diversity observed in this study suggests that the freshwater lake could serve as a reservoir for multiple strains of *A. veronii* that are pathogenic to humans.

### Global population structure of *A. veronii*

**Fig 2:**
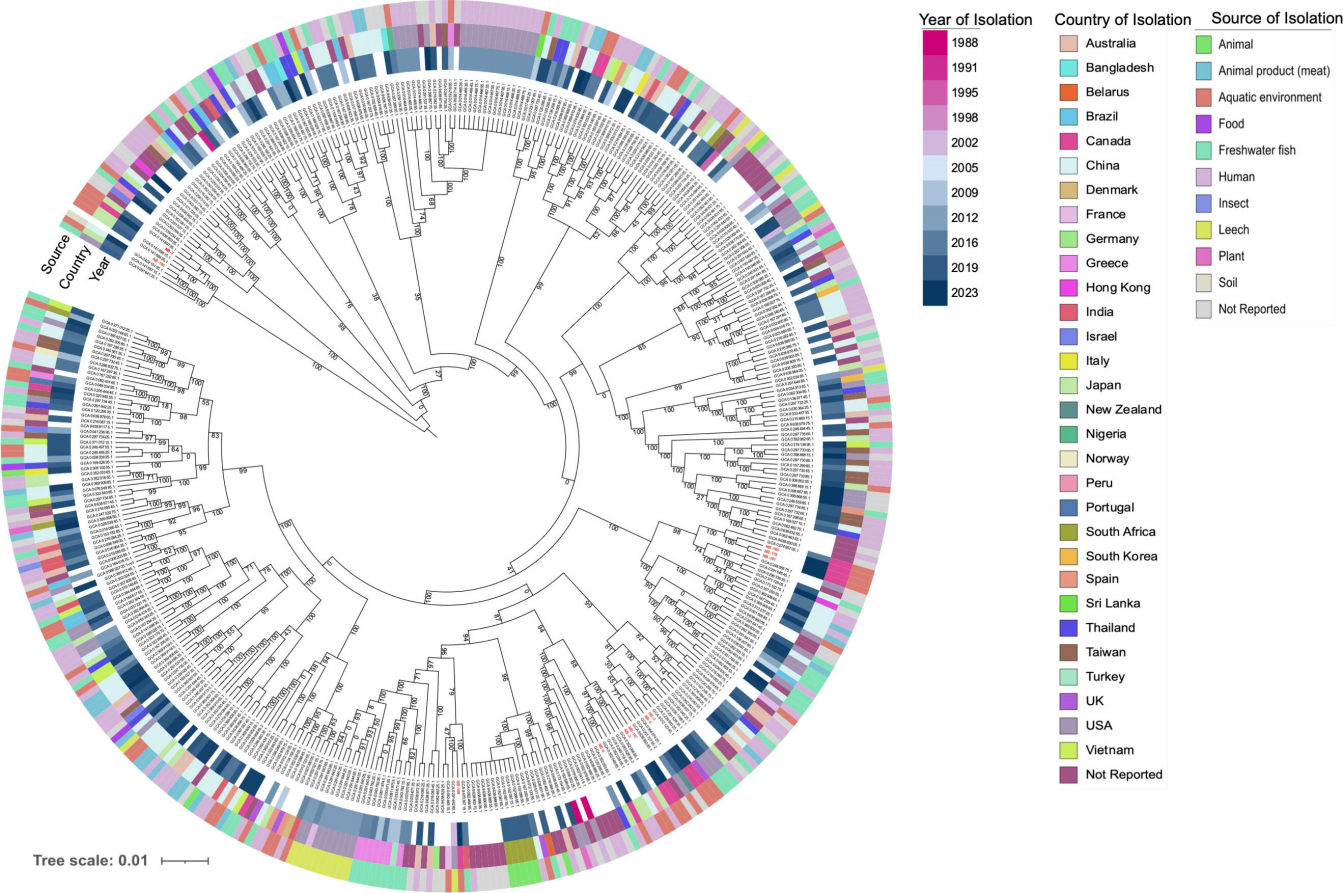
Core-genome-based maximum-likelihood tree of global *A. veronii* from different sources. Each node represents a strain. To construct the phylogeny, pangenome was generated from the annotated genomes using Roary v3.13.0. SNPs within the core-genome alignment were extracted using SNP-sites v2.5.1 using GCA_008693705.1 as a reference. The concatenated core-genome-based SNPs were used to construct a phylogenetic tree using FastTree. The general time reversible model was performed with 1,000 bootstrap resampling for node support. *A. allosaccharophila* (NB-7) and *A. caviae* (NB-180) were outgroups used for re-rooting the tree. The figure was generated using iTOL (https://itol.embl.de). *Aeromonas* isolates recovered in this study were labeled in red.

**Fig 3:**
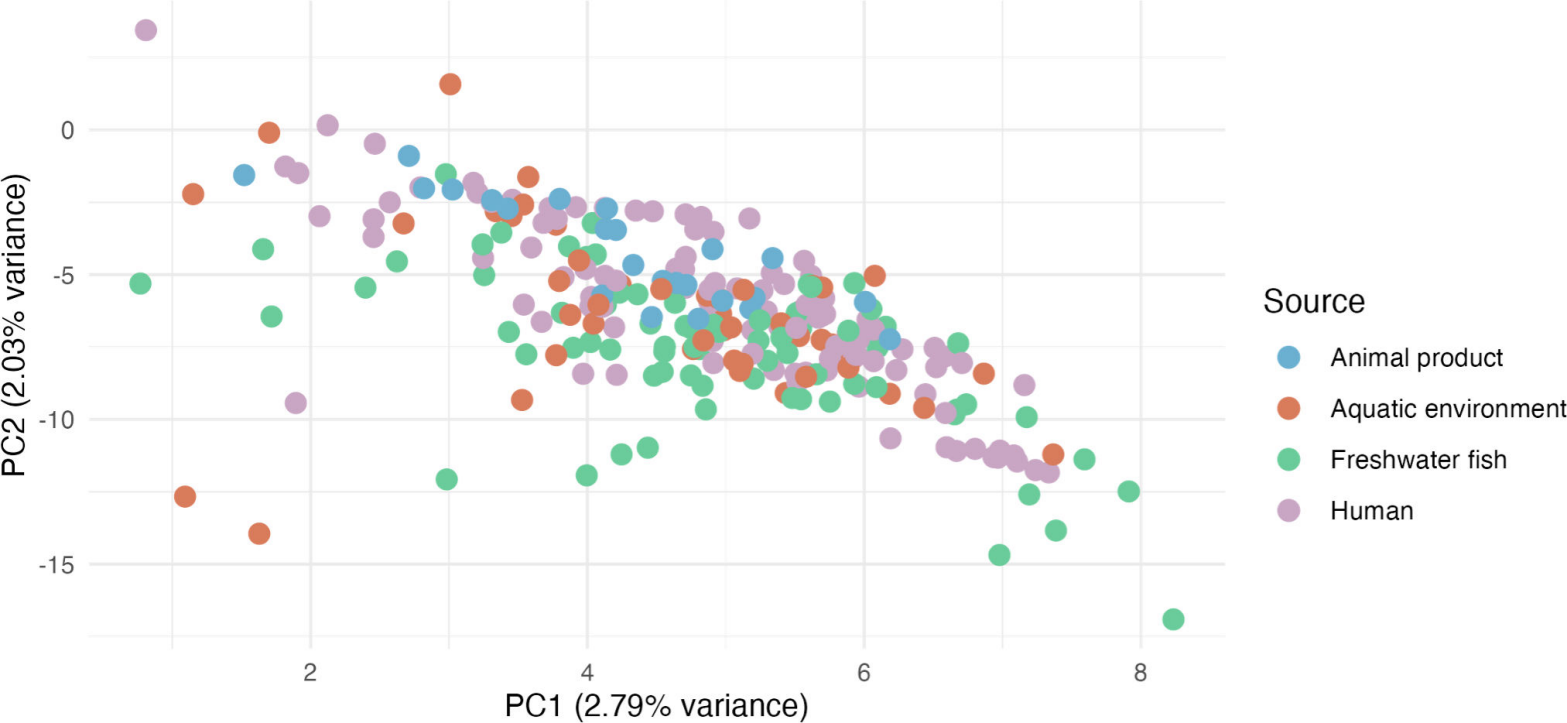
Assessment of the global *A. veronii* pangenome reveals limited clustering based on source in gene content variation. The principal component analysis (PCA) plot was generated from the pangenome (gene presence/absence) data, produced by Roary, and the associated source information. Each point on the plot represents an individual genome, positioned according to the principal components that account for the most variance in gene presence/absence data. Points are colored based on the source of isolation, with the spread of nodes indicating differences in gene content across genomes from various sources. The lack of distinct source-based clusters suggests that the variation in gene content among the genomes is not strongly correlated with their source of isolation.

To assess the genetic relatedness of the sequenced isolates with global *A. veronii*, genomes and the associated metadata of 406 *A*. *veronii* deposited in the *RefSeq* database (accessed on 13 July 2024) were downloaded and re-annotated (see Methods). The 406 genomes were recovered from 30 different countries located in six continents between 1988 to 2023 from eight different sources including human, animal, aquatic environment, freshwater fish, among others (Table S3). The pangenome size of the 406 *A*. *veronii* genomes together with the sequenced isolates (*n* = 9) yielded 54,993 genes. A total of 2,126 core genes, defined as genes present in ≥95% of the genomes in the collection, were identified, whereas the shell and cloud genes totaled 2,630 and 50,237, respectively. The core-genome SNP-based phylogeny was constructed using *A. allosaccharophila* as outgroup to root the phylogenetic tree. The sequenced *A. veronii* isolates compared with global *A. veronii* species showed high genetic diversity, which facilitated the clustering of the isolates into distinct clades (Fig. 2). Isolates sequenced in this study were clustered into distinct subclades, suggesting that they are distantly related to other global isolates. However, isolates from Turkey and Greece recovered in fish from different time points (2009, 2015, and 2016) were clustered together, a phenomenon that could suggest dissemination of *A. veronii* strains. Strain NB-188 belonged to the same subcluster as an isolate recovered from a leech in the USA in 2002. Although global *A. veronii* species were distantly related, we could still observe a mixture of isolates from different countries and sources within some clusters. Overall, *A. veronii* from different environments may have genetic signatures unique to pathogenic strains of this bacterium. This could also be important to determine or predict the source of isolates found in any matrix. However, the pan-GWAS approach did not yield any gene with strong association with source of isolation (Fig. 3).

### Stress response genes among the sequenced isolates and in global *A. veronii*

**Fig 4:**
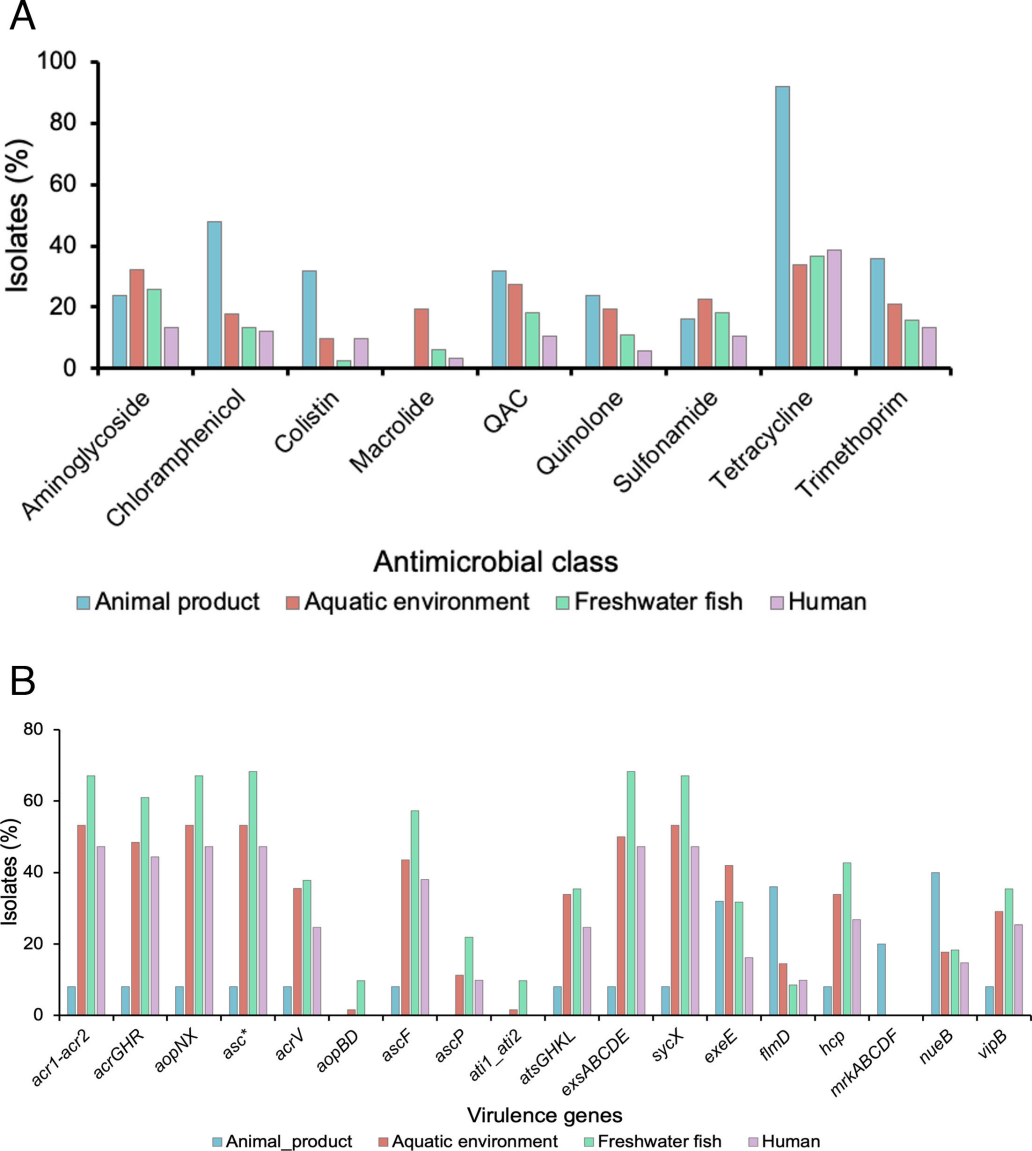
Distribution of genes encoding resistance to different classes of antimicrobials (**A**) and virulence (**B**) across various sources in a global collection of *A. veronii* genomes

Genes encoding resistance to β-lactams were detected in all the isolates sequenced in this study. Different alleles of *cphA* (*cphA3, cphA4,* and *cphA7*) gene, which belonged to the subclass B2 metallo-β-lactamase that encodes resistance to carbapenem antibiotics were detected in all *A. veronii* isolates and in *A. allosaccharophila. OXA-912* that encodes resistance to penams, cephalosporins and carbapenems, and *cphA3* genes were predominant in the collection (Fig. 1). The rescreening of global *A. veronii* genomes for AMR genes revealed that these β-lactam resistance genes were ubiquitous in this bacterium (Fig. S1; Table S4). Genes encoding resistance to nine other classes of antibiotics were detected in the global collection, with tetracycline resistance genes being the second most prevalent after β-lactam resistance genes, found in 35% of the collection. The prevalence of resistance genes for the other classes (including aminoglycosides, chloramphenicol, colistin, macrolides, quaternary ammonium compounds, quinolones, sulfonamides, and trimethoprim) ranged from 7% to 18% (Fig. S1; Table S4). Of note, some AMR genes were enriched in a few isolates from different sources. Genes encoding resistance to colistin, chloramphenicol, trimethoprim, and tetracycline were enriched in isolates from animal products. In contrast, macrolide resistance genes show a higher prevalence in isolates from the aquatic environment. Freshwater fish and human sources exhibit a more balanced distribution of these resistance genes, with no single class being significantly dominant (Fig. 4A). Overall, these results suggest that specific environmental and/or anthropogenic factors may influence the prevalence of AMR genes in *A. veronii* from different sources.

The detection of virulence genes using a gene homology approach (see Methods) and a curated virulence gene database (VFDB [36, 37]) detected 8 to 48 virulence genes in each *A. veronii* isolate sequenced in this study. Relative to isolates predicted as human pathogens that contained 41–48 virulence genes (except NB-3), all the *A. veronii* predicted as non-human pathogens carried less virulence genes (≤9 virulence genes). Human and non-human pathogenic strains of *A. veronii* differed in terms of their virulence gene content. Although the flagellar and type IV pili-associated genes involved in biofilm formation (18, 37) were detected in all isolates, type III secretion system (T3SS)-associated genes were detected only in the isolates predicted as human pathogens (Fig. 1). The assessment of virulence genes in the global collection of *A. veronii* revealed a slight differential distribution of T3SS-associated genes and other virulence factors across isolates from different sources with freshwater fish isolates carrying more virulence genes compared to those from other sources (Fig. 4B). Isolates from animal products carried the least number of virulence genes except for the *nueB* gene. Also, the *mrkABCDF* operon, which encodes type III fimbriae and involved in biofilm formation on biotic and abiotic surfaces in *Klebsiella* and *Citrobacter* (36, 37), was exclusively found in isolates recovered from animal products (Fig. 4B). This overall pattern suggests a somewhat source-dependent variation in virulence gene content, highlighting the potential for distinct pathogenic profiles in a few *A. veronii* isolates from different environments.

### Characterization of mobile genetic elements among the sequenced isolates and in global *A. veronii*

**Fig 5:**
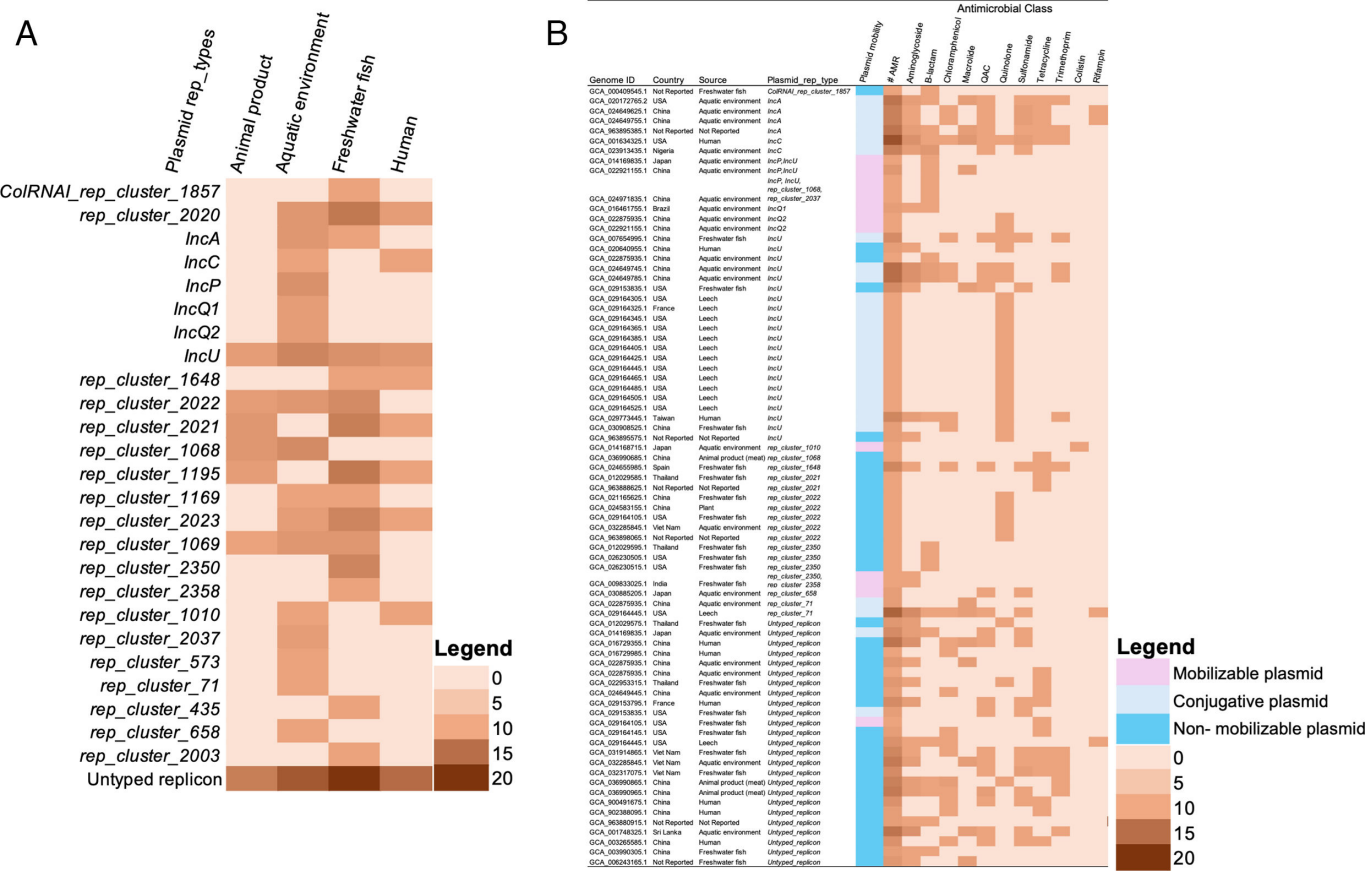
Source-dependent distribution of plasmid types (**A**) and plasmids encoding resistance to multiple classes of antimicrobials (**B**) in a global collection of *A. veronii*. The color shades reflect the relative abundance of each plasmid type (**A**) or the number of antimicrobial resistance genes (**B**).

Plasmids were not detected in any of the *A. veronii* isolates sequenced in this study. However, rescreening of global *A. veronii* genomes identified 25 different known plasmid types in 32% (*n* = 130/406) of the genomes, with the IncU plasmid being the most predominant type in the collection (Table S5). Although isolates recovered from the aquatic environment and freshwater fish had the highest diversity of plasmid types, the Inc plasmid types (IncC, IncP, IncQ1, IncQ2, IncU) were particularly enriched in isolates from the aquatic environment (Fig. 5A). Of note, 16% (*n* = 67/406) of the genomes carried plasmids with genes encoding resistance to at least one antimicrobial class, the great majority (*n* = 39/67) of which were non-mobilizable, suggesting that they could be cryptic plasmids. The remaining few that carried mobilizable/conjugative plasmids were predominantly recovered from the aquatic environment (Fig. 5B). Of particular interest was a previously described IncC plasmid (54) that carried 15 AMR genes that encode resistance to nine different classes of antimicrobials including aminoglycoside, chloramphenicol, macrolides, sulfonamide, among others (Fig. 5B). The analysis of the other genes in this genetic element showed that it contains phage genes. A further screening of the plasmid through the phage detection pipeline revealed an intact phage region within this plasmid, suggesting that this genetic element is indeed a phage-like plasmid (55, 56).

The detection and characterization of phage regions in the isolates sequenced in this study yielded 13 unique intact phages, among which four were predicted to be virulent phages (43). The completeness of all intact phage sequences was determined to be between 50% and 100% by CheckV (41). The phages were classified by PhaGCN (42) as Peduoviridae (*n* = 10), Chaseviridae (*n* = 1), and two others unidentified according to the International Committee on Taxonomy of Viruses (ICTV) classification (57). In addition to *A. veronii* being predicted as host of the phages, other species of *Aeromonas* (*A. australiensis*, *A. diversa*, *Aeromonas* sp.) and *Serratia marcescens* could also serve as their hosts as determined by CHERRY (40, 44), suggesting that these phages could infect multiple hosts (Table 2). Of note, the two pairs of isolates (NB-2/NB-4 and NB-178/NB-181) that were highly genetically related by SNP had the same phage content. No antibiotic resistance, toxin, or related genes were detected in the intact phages. All the intact phages detected were screened for TSPs using TSPDB that contains 8,105 TSPs (49), but none was found.

**TABLE 2:** Features of intact phages detected in *A. veronii* sequenced in the study

Isolate ID	Intact phage region	Length (bp)	Phage lifestyle	Phage family	Host	Gene count	Viral genes	Host genes	% Phage completeness
NB-178	NB-178_00010_193626_97002–124946	27,945	Temperate	Peduoviridae	*S. marcescens*	34	20	4	59.34
NB-178	NB-178_00010_193626_138510–165106	26,597	Virulent	Peduoviridae	*A. diversa*	29	21	2	64.38
NB-180	NB-180p_1:101319–141612	40,294	Virulent	Unknown	*A. veronii*	39	24	2	67.14
NB-180	NB-180p_1:207148–238244	31,097	Temperate	Peduoviridae	*A. australiensis*	41	17	1	84.82
NB-185	NB-185p_1:196668–255973	59,306	Virulent	Chaseviridae	*Aeromonas* sp. *DNP9*	82	34	2	97.53
NB-185	NB-185p_14:30853–63241	32,389	Temperate	Peduoviridae	*A. veronii*	45	26	0	95.3
NB-185	NB-185p_3:133090–168585	35,496	Virulent	Unknown	*A. veronii*	56	29	0	100
NB-188	NB-188_00002_602127_302137–337616	35,480	Virulent	Peduoviridae	*A. australiensis*	45	18	0	96.81
NB-188	NB-188_00010_161842_8413–46184	37,772	Temperate	Peduoviridae	*A. veronii*	56	30	1	100
NB-2	NB-2_00001_820342_705754–750131	44,378	Temperate	Peduoviridae	*A. veronii*	70	35	5	92.49
NB-2	NB-2_00009_103898–141465	37,568	Temperate	Peduoviridae	*Aeromonas* sp. *L_1B5_3*	56	33	1	100
NB-3	NB-3_00002_655010_341482–372468	30,987	Temperate	Peduoviridae	*A. australiensis*	41	18	0	84.52
NB-7	NB-7_00020_76813_28309–66369	38,061	Temperate	Peduoviridae	*A. australiensis*	49	24	1	100

### Biosynthetic gene cluster profile in *A. veronii*

**Fig 6:**
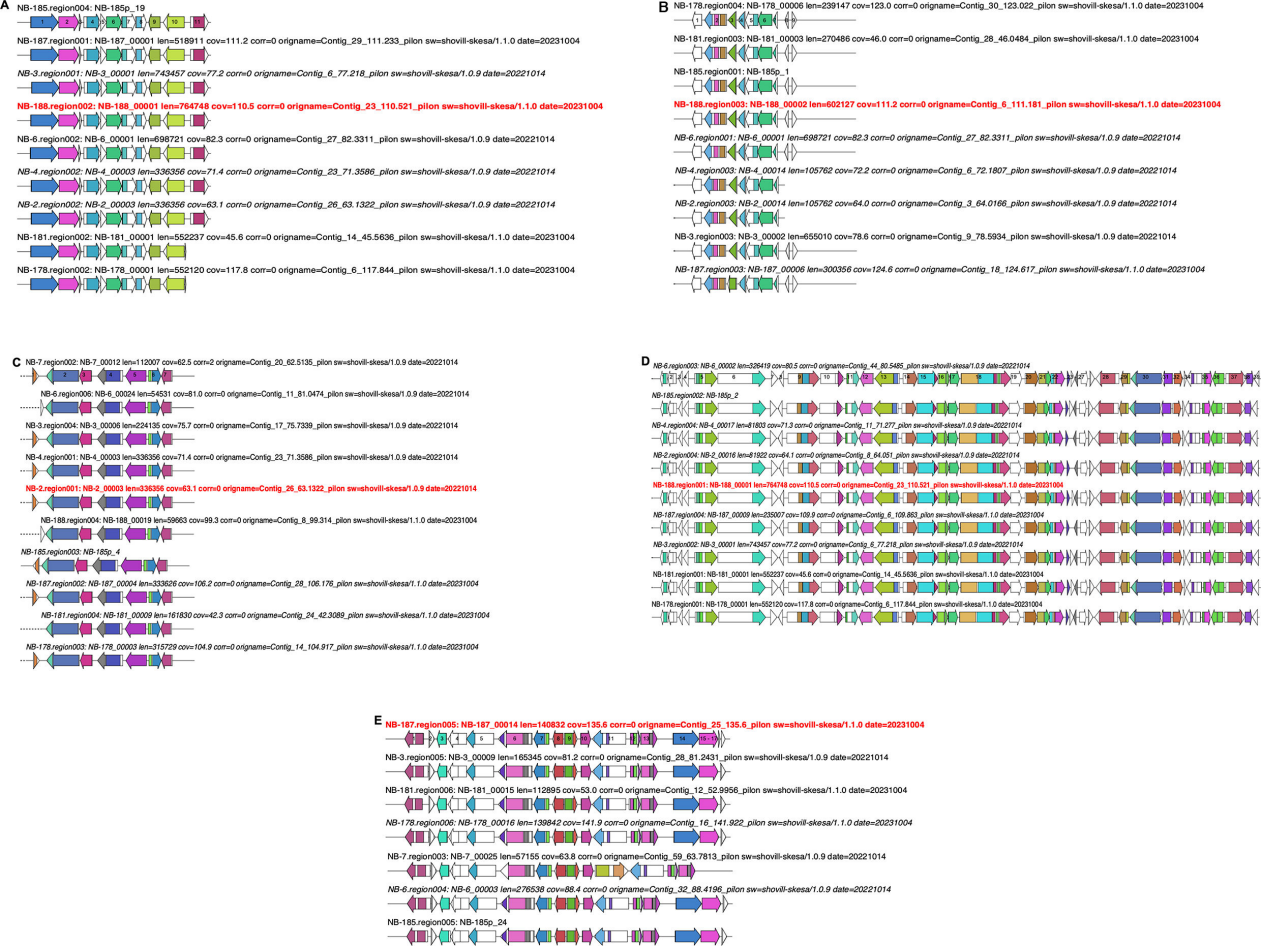
Novel biosynthetic gene clusters identified in *A. veronii* sequenced. (**A)** RiPP BGC with a low similarity score of 0.17 to angustmycin A/B/C in *S. angustmyceticus* (accession MZ151497.1). (**B**) RiPP gene cluster with a low similarity score of 0.04 pseudopyronine A/B found in *P. putida* (accession KT373879.1).** (C)** RiPP gene cluster detected in *A. veronii* and *A. allosaccharophila* with a low similarity score of 0.08 to pseudopyronine A/B found in *P. putida* (accession KT373879.1). (**D**) NRPS BGC with a 0.9 similarity to enterobactin in *Pseudomonas* sp. J465 (accession GQ370384.1). (**E)** Homoserine lactone detected in *A. veronii* and in *A. allosaccharophila* with a low similarity score of 0.14 to thioguanine found in *E. amylovora* CFBP1430 (accession number: NC_013971.1).

**Fig 7:**
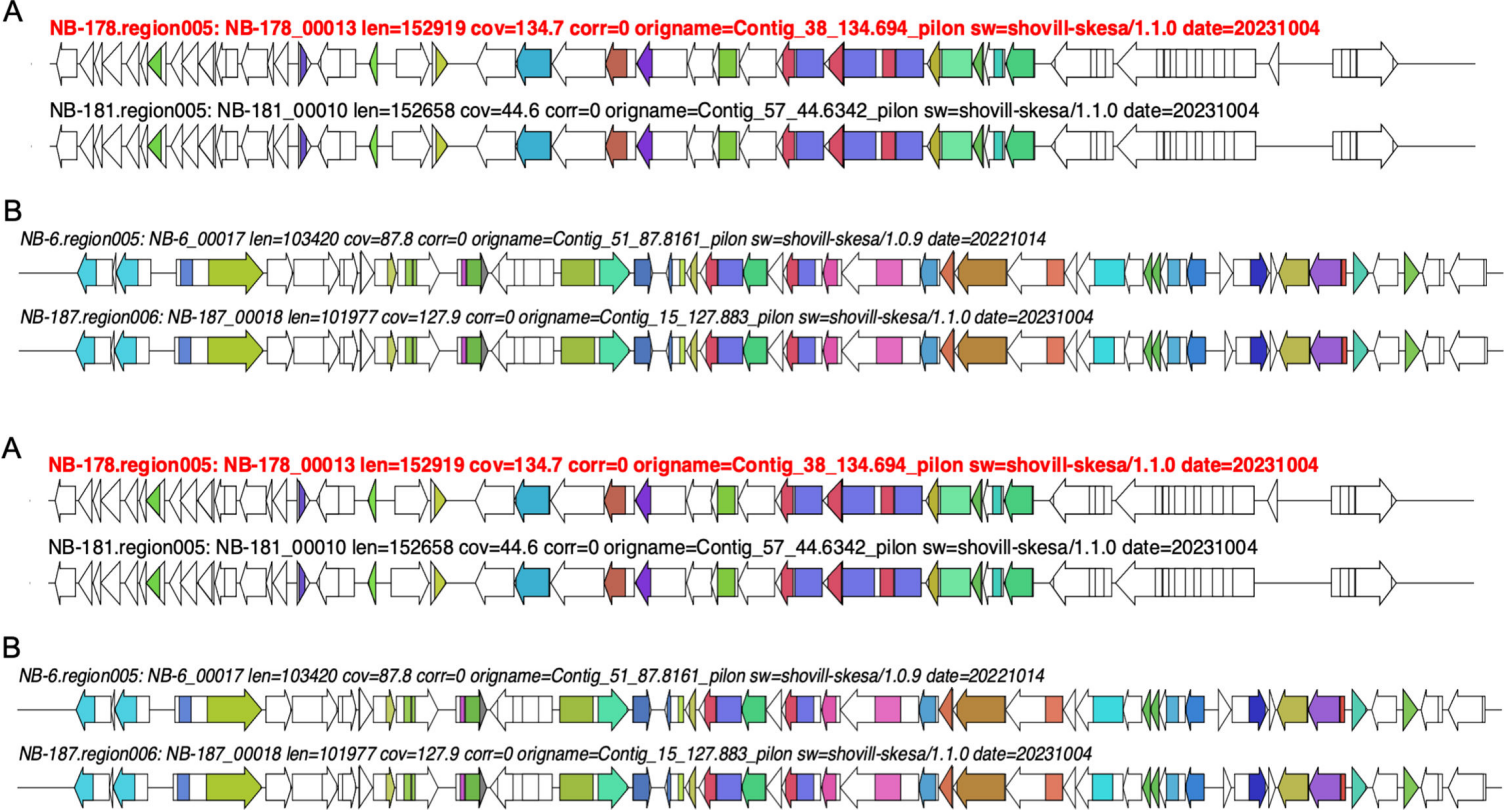
Biosynthetic gene clusters identified in *A. veronii* predicted to be human or non-human pathogens. (**A**) Aryl polyene gene cluster detected in *A. veronii* isolates predicted to be a non-human pathogen with a similarity score of 0.26 to bacilysin found in *Bacillus* sp. CS93 (accession number: GQ889493.1). (**B**) Aryl polyene gene cluster detected in *A. veronii* isolates predicted to be a human pathogen with a similarity score of 0.44 to aryl polyene found in *X. doucetiae* (accession number: NZ_FO704550.1).

The 53 biosynthetic gene clusters (BGCs) identified in all sequenced *Aeromonas* species were categorized into eight BGC families using sequence similarity network analysis with BiGSCAPE (58). The ribosomally synthesized and post-translationally modified peptides (RiPPs) were the most predominant BGC class, consisting of three gene families. In contrast, the non-ribosomal polyketide synthase (NRPS) included only one gene family. The remaining gene families were classified as “others” and included homoserine lactone (*n* = 2) and aryl polyene (*n* = 2). The three RiPPs detected were unique and conserved within the collection but exhibited low similarity scores to previously described BGCs. For example, RiPP-1 (Fig. 6A), comprising 11 open reading frames (ORFs), had a similarity score of 0.17 to angustmycin A/B/C (BGC0002621) described in *Streptomyces angustmyceticus* (accession MZ151497.1) (59). Meanwhile, RiPP-2 and RiPP-3 (Fig. 6B and C), consisting of nine and seven ORFs, respectively, had similarity scores of ≤0.08 to pseudopyronine A/B (BGC0001285) described in *Pseudomonas putida* (accession KT373879.1) (60). Notably, RiPP-3 was also detected in *A. allosaccharophila* (NB-7), indicating that this BGC is not exclusive to *A. veronii* (Fig. 6C). The identified NRPS had the highest similarity score of 0.9 to enterobactin (BGC0000343) previously described in *Pseudomonas* sp. J465 (accession GQ370384.1) (61). This BGC was conserved in the *A. veronii* sequenced (Fig. 6D). Further analysis of global *A. veronii* genomes confirmed that this BGC was conserved not only in this collection but also in all publicly available *A. veronii* genomes. A BGC encoding homoserine lactone, predominant in *A. veronii* (*n* = 6/9), was also detected in *A. allosaccharophila*. This BGC had a low similarity score (0.14) to thioguanine (BGC0001992) in *Erwinia amylovora* CFBP1430 (accession number: NC_013971.1) (62). Notably, a pair of *A. veronii* strains—one pathogenic (NB-6/NB-187) and one non-pathogenic to humans (NB-178/NB-181)—carried unique BGCs encoding aryl polyene (Fig. 7A and B). The pathogenic pair consisted of 17 ORFs with a similarity score of 0.44 to aryl polyene (BGC0002008) described in *Xenorhabdus doucetiae* (accession NZ_FO704550.1) (63), whereas the non-pathogenic pair contained 37 ORFs with a similarity score of 0.26 to bacilysin (BGC0000888) described in *Bacillus* sp. CS93 (accession number: GQ889493.1) (64). Overall, *A. veronii* harbored putative unique BGCs that exhibited low similarity scores to previously described compounds.

## DISCUSSION

Aquatic ecosystems are continually impacted by anthropogenic activities, making the microbial quality and safety of these water bodies, especially those used for recreational activities, paramount for public health (1). In this study, we assessed the presence of *A. veronii* in a recreational lake and determined the extensive genomic features of the isolates regarding their population structure and the genomic characterization of stress response genes, mobile genetic elements, and other gene content such as biosynthetic gene clusters that confer uniqueness to different *A. veronii* strains. We inferred the global population structure of *A. veronii* by assessing the genetic relatedness of the isolates sequenced with previously sequenced strains in public databases.

In the past decade, WGS has become the gold standard method for species identification, complementing existing biochemical-based methods (65). In this study, the WGS-based approach identified isolates as *A. veronii, A. caviae*, and *A. allosaccharophila*, whereas the biochemical identification system misidentified all isolates as either *A. sobria* or *A. hydrophila*. Misidentification of species of environmental bacteria by biochemical approaches is not uncommon (65). Studies comparing biochemical-based bacterial species identification systems to WGS have shown that species misidentification can vary by species and is common in specific bacteria, including *Pseudomonas fluorescens*, *P. putida* (65), and *Enterococcus faecalis* (66).

*A. veronii* strains sequenced exhibited different pathogenic potentials, with the majority (67%, *n* = 6/9) predicted to be pathogenic to humans and possessing a similar virulence determinant profile, including T3SS. T3SSs are crucial virulence mechanisms that allow bacteria to inject effector proteins directly into the host cell cytoplasm. The activity of T3SSs closely correlates with infection progression and outcome in various infection models, and its presence is considered a general indicator of virulence in *A. veronii* (67–69). The detection of human pathogenic *A. veronii* in this study, along with other clinically relevant pathogens such as *B. anthracis* (21) and *V. cholerae* (22) in this recreational lake from previous studies, emphasizes the crucial role aquatic ecosystems play in disseminating pathogens. The recreational use of this water could pose a continuous risk to public health, serving as a reservoir and facilitating the transmission of waterborne diseases. This also underscores the significance of monitoring aquatic environments as reservoirs for pathogenic bacteria.

There was a high genetic diversity among the nine *A. veronii* isolates sequenced, including the identification of novel sequence types and alleles. Although some strains were indistinguishable by SNPs, others were genetically distant. This finding could imply that different *A. veronii* strains may have been introduced into the lake multiple times from various sources such as resident freshwater fish, domestic animals, and environmental samples (5). The integration of genomic data from the *A. veronii* isolates sequenced with global strains revealed that isolates from single sites formed smaller groups within the phylogeny. Interestingly, one isolate from this study (NB-188) was nested with an isolate recovered from leech in a crop field in the USA. A previous study assessing the core-genome-based phylogenetic analysis of *A. veronii* genomes deposited in NCBI from 18 countries revealed a high genetic diversity (5). The admixture of *A. veronii* strains from different sources was observed, suggesting a lack of source- and timepoint-based clustering in the *A. veronii* population. However, strains from a single site tend to form small groups within the phylogenetic clusters. These observations concur with our findings. The genetic diversity observed in *A. veronii* reinforces the importance of continuous genomic surveillance to monitor the emergence and spread of virulent and/or resistant strains.

The AMR determinant profile observed in the isolates sequenced in this study was comparable and included only chromosome-borne genes encoding resistance to β-lactams. The widespread presence of β-lactam resistance genes, including those conferring resistance to carbapenems, is a known phenomenon in the *A. veronii* population (10). Although these genes were chromosomal with no close proximity to mobile genetic elements, their spread to other strains or bacterial species is not entirely unlikely as bacterial cell lysis could release DNA into the environment where it could be taken up by other strains or bacterial species through the process of transformation. Indeed, natural transformation has been described as a common mechanism of horizontal gene transfer (HGT) among *Aeromonas* species, including *A. veronii. Aeromonas* species are capable of competence and transformation (70). In addition, *A. veronii* is known to easily acquire and exchange AMR genes (7, 20, 71). Although there was a low occurrence of AMR in *A. veronii* sequenced in this study, Lake Wilcox is a potential reservoir for AMR genes encoding resistance to multiple antibiotics as evidenced by results from previous studies on the lake where other bacterial species isolated from the lake carried multiple AMR genes (21, 22). The assessment of AMR gene profiles in global *A. veronii* revealed variability in isolates across different sources, highlighting the influence of environmental and host-specific factors on the emergence and spread of AMR in this bacterial population (72–74). More so, the detection of virulence genes in the isolates sequenced in this study, particularly those associated with the T3SS in human pathogenic strains, further emphasizes the potential health risks posed by these bacteria. The T3SS appeared to be predominant in global isolates recovered from the aquatic environment, freshwater fish, and human but less prevalent in animal products. Although T3SS is linked to severity of disease by Gram-negative bacterial pathogen such as *Aeromonas* (75), they have been documented to play a key role in the bacterial adaptation to changing environmental conditions (68, 75–77). They have also been noted to be dynamic and constantly exchanging components and facilitating interactions between other microorganisms including fungi, depending on the ecological and evolutionary needs (68, 75–77). The predominance of the *mrkABCDF* operon, which encodes type III fimbriae involved in biofilm formation on diverse surfaces (36, 37), in isolates recovered from animal products is notable. This operon may contribute to the persistence of *A. veronii* in animal processing facilities, potentially leading to microbial contamination of animal products (13, 78).

The mobilome is known to facilitate gene gain and loss, a phenomenon that plays a crucial role in bacterial evolution and ecological adaptation, and a probable change in bacterial fitness (79, 80). This change can contribute to the emergence of divergent bacterial populations with unique features, including higher pathogenic potential (79, 81, 82). Although we did not detect plasmids in our isolates, global data revealed their presence and association with multidrug resistance genes. Of note was a phage-like plasmid that was found to contain genes encoding resistance to nine classes of antimicrobials including heavy metal (mercury) (54). Phage-like plasmids are plasmids that share structural and functional features of phage and enable them to replicate and facilitate the HGT of genes (including AMR) between bacteria (55, 83, 84). Other MGEs including prophages and insertion sequences were identified. The majority of the intact prophages were identified as P2-like phages (Peduoviridae) (57), and a few of them were predicted to have multiple host bacterial species. This observation is interesting and could suggest a broad host range of these phages, which could have applications in biocontrol (85–87), but further studies on the host range of these phages would be needed to ascertain this. Another factor that contributes to the rapid evolution and ecological adaptation and that could influence the pathogenicity of bacterial species is BGCs that encode the production of various secondary metabolites (60, 88, 89). This phenomenon is seldom studied in *A. veronii*.

In this study, we found a high abundance of novel BGCs and identified unique NRPS and RiPP that were conserved in *A. veronii*. Notably, NRPS with high similarity (0.9) to enterobactin was found in *Pseudomonas* sp. J465 (53), which mediates high affinity for iron acquisition in stringent conditions (90, 91). Angustmycin A/B/C (51) and pseudopyronine A/B (52) homologs were found to be conserved in *A. veronii*. These RiPP products encode antimicrobial properties and contribute to the survival of their producers in their ecological niche (59, 92). These conserved clusters could be promising genomic markers for typing *A. veronii*. Of note, a bacilysin homolog gene (64) was detected in a pair of non-human pathogenic strains. Bacilysin is an antimicrobial dipeptide produced by *Bacillus* spp. that exhibits antagonistic activity against both Gram-negative and Gram-positive bacteria (64, 93, 94). Further studies would be required to decipher the antimicrobial activity of the bacilysin homolog identified in this study against human pathogenic strains of *A. veronii* and other pathogens, as well as their mechanism of actions.

### Conclusion

The study presents a genomic analysis of *A. veronii* strains isolated from a freshwater lake, defines the population structure, and characterizes the genetic factors associated with stress and ecological adaptation. A significant finding is the pathogenic potential of *A. veronii* to humans that underscores the public health implications, especially considering the recreational use of the lake. The MGEs identified that could contribute to the genetic diversity, adaptability, and pathogenicity to human, as well as the role of *A. veronii* as a reservoir for AMR genes, while the BGCs identified presents opportunities for the discovery of novel bioactive compounds. Overall, this study not only contributes to our understanding of the genetic diversity and ecological dynamics of *A. veronii* but also highlights the potential public health risks and AMR reservoir role of this bacterium. It underscores the need for continuous surveillance for pathogens in aquatic ecosystems.

## Data Availability

The whole-genome sequences reported in this study were deposited at DDBJ/ENA/GenBank under the BioProject accession numbers PRJNA893208. The raw sequence reads and genome assembly accession numbers are listed in Table 1. In addition, accession numbers and associated metadata of genomes retrieved from NCBI are listed in Table S3.

## SUPPLEMENTAL MATERIAL

Table S1 spectrum.01171-24-s0001.csv Summary of the novel alleles and sequence types identified in *Aeromonas* isolates.

Table S2 spectrum.01171-24-s0002.csv Genetic distance among *Aeromonas veronii* isolates sequenced in this study.

Table S3 spectrum.01171-24-s0003.csv List of accession number and associated metadata of publicly available *Aeromonas veronii* genomes.

Table S4 spectrum.01171-24-s0004.csv Antimicrobial resistance genes in a global collection of *Aeromonas veronii*.

Table S5 spectrum.01171-24-s0005.csv Distribution of plasmid types in a global collection of *Aeromonas veronii*.

Fig S1 spectrum.01171-24-s0006.tiff Distribution of plasmid types in a global collection of *Aeromonas veronii*.

ASM does not own the copyrights to Supplemental Material that may be linked to, or accessed through, an article. The authors have granted ASM a non-exclusive, world-wide license to publish the Supplemental Material files. Please contact the corresponding author directly for reuse.
