# Supplementary figures and images for: Phylogenomics of novel clones of Aeromonas veronii recovered from a freshwater lake reveals unique biosynthetic gene clusters
Source: Microbiol Spectr. 2024 Nov 8;12(12):e01171-24. doi: 10.1128/spectrum.01171-24 (PMC11619367; doi:10.1128/spectrum.01171-24)

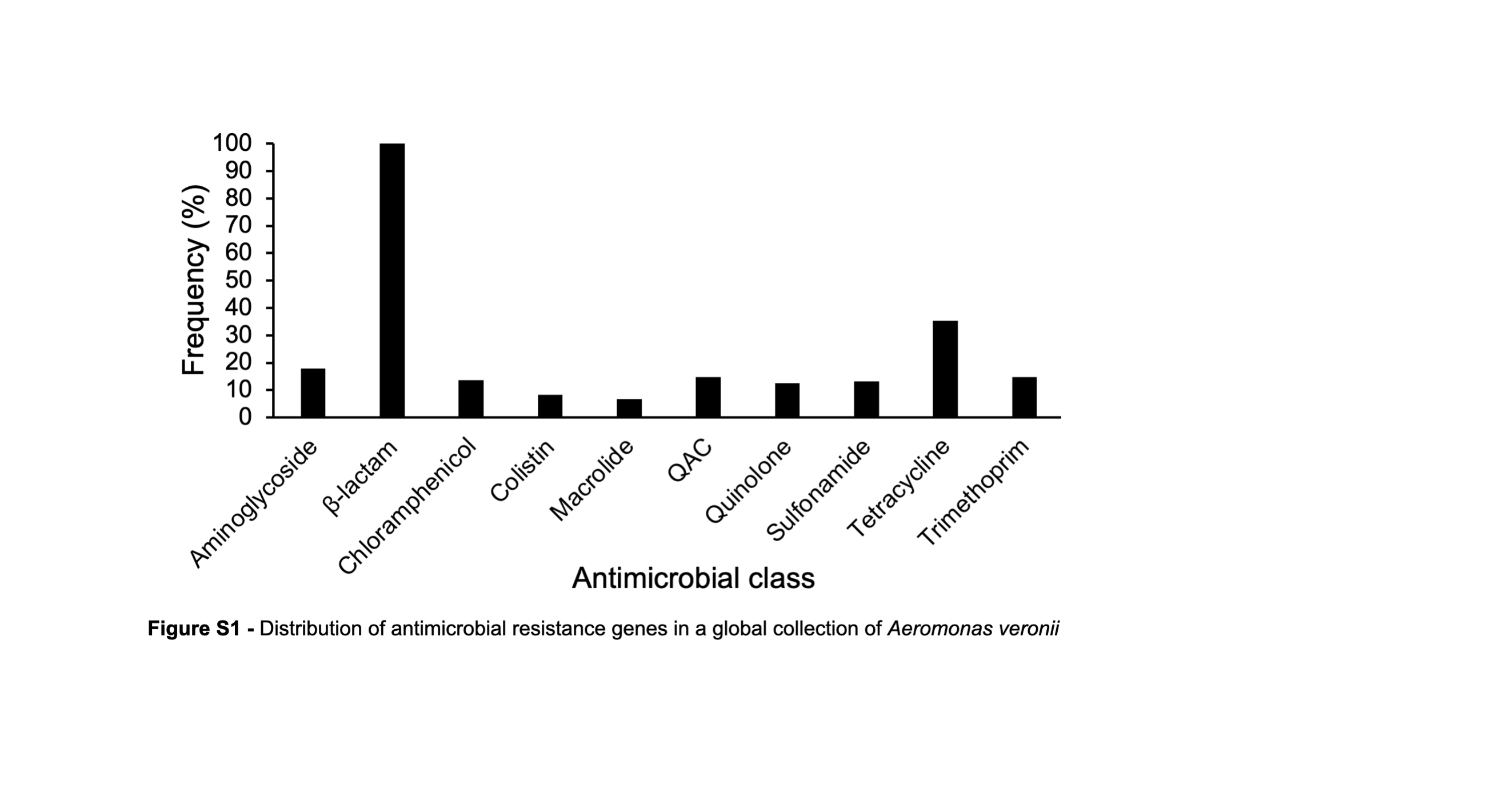

Supplement: Fig S1 — Distribution of plasmid types in a global collection of Aeromonas veronii. [file spectrum.01171-24-s0006.tiff]
